# Supplementary material for: SpermQ–A Simple Analysis Software to Comprehensively Study Flagellar Beating and Sperm Steering
Source: Cells. 2018 Dec 26;8(1):10. doi: 10.3390/cells8010010 (PMC6357160; doi:10.3390/cells8010010)
Supplement: Supplementary file 1 [file cells-08-00010-s001.pdf]

SpermQ - a simple analysis software to comprehensively study flagellar beating and sperm steering

Supplementary Figures

Jan N. Hansen[1], Sebastian Raßmann[1], Jan F. Jikeli[1]*, and Dagmar Wachten[1,2]*

[1] Institute of Innate Immunity, Biophysical Imaging, University Hospital Bonn, University of Bonn, 53127 Bonn, Germany

[2] Center of Advanced European Studies and Research (caesar), Molecular Physiology, 53175 Bonn, Germany

* equally contributed

# Correspondence: dwachten@uni-bonn.de, +49-228-9656-311

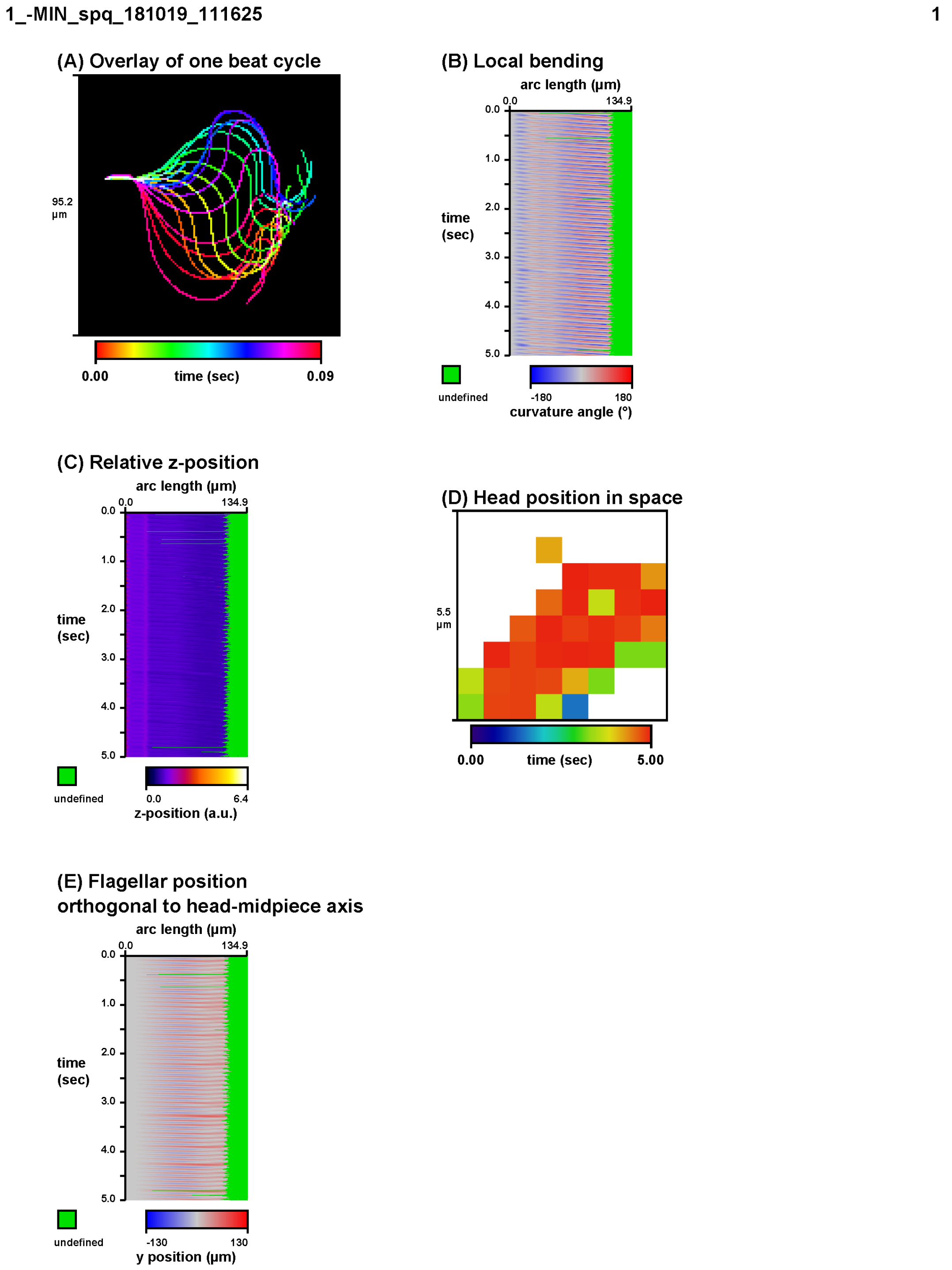

Figure S1: Exemplary output file generated by *SpermQ Evaluator* for a head-tethered mouse sperm – Page 1. For each analyzed sperm, *SpermQ Evaluator* creates a two-pages PDF with plots of the most important parameters. Page 1 of this output file contains the image name and page number on top, (A) a time-projection image of the frames belonging to the first beat cycle, (B) the kymograph of the curvature angle, (C) the kymograph of the relative z-position, (D) a time projection of the head position in space, which – if the sperm cell is freely swimming – shows the trajectory of the sperm cell, and (E) the kymograph of the y position relative to the head-midpiece axis.

1_-MIN_spq_181019_111625 2

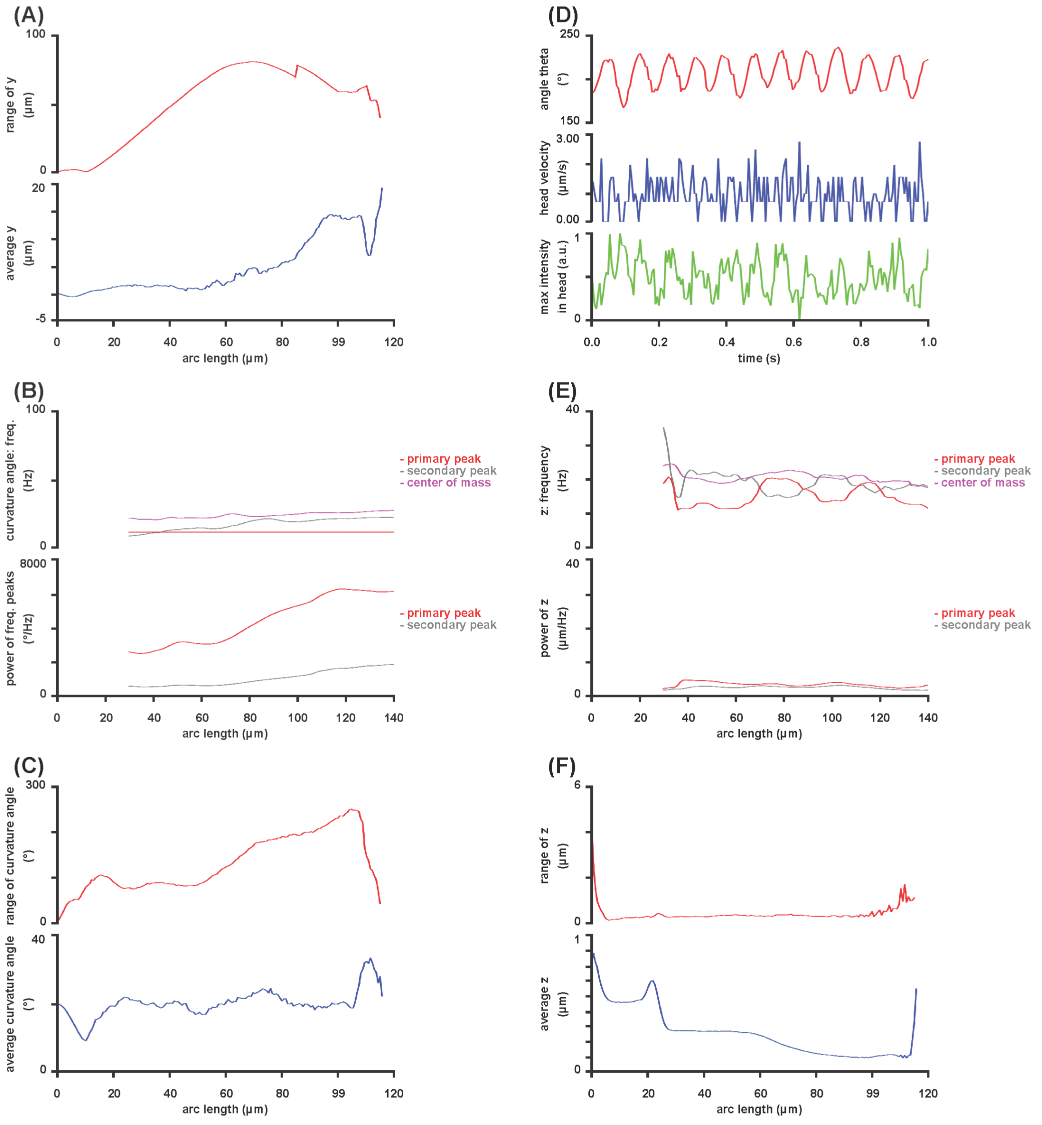

(A) upper panel: beat amplitude compared to head-midpiece axis, bottom panel: asymmetry compared to head-midpiece axis
(B) local beat frequency
(C) upper panel: local bending amplitude, bottom panel: local bending asymmetry
(D) upper panel: orientation of head-midpiece axis in space, middle panel: velocity of head in space, bottom panel: rolling
(E) local beat frequency in z
(F) upper panel: beat amplitude in z, bottom panel: asymmetry in z

This file has been created by the SpermQ_Evaluator tool using the Apache PDFBox, JFreeChart and ImageJ packages for Java. The poject is available at https://github.com/hillimaging/SpermQ_Evaluator

Figure S2: Exemplary output file generated by *SpermQ Evaluator* for a head-tethered mouse sperm – Page 2. For each analyzed sperm, *SpermQ Evaluator* creates a two-pages PDF with plots of the most important parameters. Page 2 of this output file contains the image name and page number on top and plots of (A) the range and average of the y position over time, of (B) the frequency results for the parameter curvature angle, of (C) the average and range of the curvature angle over time, and of (D) the angle theta, head velocity, and the maximum intensity in the head for the first analyzed second. Head velocity and the maximum intensity in the head are indicative only for freely swimming sperm. Furthermore, the page shows plots of (E) the frequency results for the relative z position, and of (F) the average and range of the relative z position over time. All plots are further explained in a legend on the bottom of the page.
